# Supplementary material for: Resolving the full spectrum of human genome variation using Linked-Reads
Source: Genome Res. 2019 Apr;29(4):635–45. doi: 10.1101/gr.234443.118 (PMC6442396; doi:10.1101/gr.234443.118)
Supplement: Supplemental Material [file supp_gr.234443.118_Supplemental_longranger-2.2.zip › longranger-2.2.tar.bz2!/lr2.2-public/lib/stan/cmdstan/stan/doc/api/html/adapt__unit__e__static__hmc_8hpp_source.html]

Stan: src/stan/mcmc/hmc/static/adapt\_unit\_e\_static\_hmc.hpp Source File


|  |  |
| --- | --- |
|  | Stan  2.5.0  probability, sampling & optimization |


- Main Page
- Related Pages
- Namespaces
- Classes
- Files

- File List
- File Members

All Classes Namespaces Files Functions Variables Typedefs Enumerations Enumerator Friends Macros Pages

- src
- stan
- mcmc
- hmc
- static

adapt\_unit\_e\_static\_hmc.hpp

Go to the documentation of this file.

1 #ifndef STAN\_\_MCMC\_\_ADAPT\_\_UNIT\_\_E\_\_STATIC\_\_HMC\_\_BETA

2 #define STAN\_\_MCMC\_\_ADAPT\_\_UNIT\_\_E\_\_STATIC\_\_HMC\_\_BETA

3

4 #include <stan/mcmc/stepsize\_adapter.hpp>

5 #include <stan/mcmc/hmc/static/unit\_e\_static\_hmc.hpp>

6

7 namespace stan {

8

9  namespace mcmc {

10

11  // Hamiltonian Monte Carlo on a

12  // Euclidean manifold with unit metric,

13  // static integration time,

14  // and adaptive stepsize

15

16  template <typename M, class BaseRNG>

17  class adapt\_unit\_e\_static\_hmc: public unit\_e\_static\_hmc<M, BaseRNG>,

18  public stepsize\_adapter {

19

20  public:

21

22  adapt\_unit\_e\_static\_hmc(M &m, BaseRNG& rng,

23  std::ostream\* o = &std::cout, std::ostream\* e = 0):

24  unit\_e\_static\_hmc<M, BaseRNG>(m, rng, o, e) {};

25

26  ~adapt\_unit\_e\_static\_hmc() {};

27

28  sample transition(sample& init\_sample) {

29

30  sample s = unit\_e\_static\_hmc<M, BaseRNG>::transition(init\_sample);

31

32  if (this->adapt\_flag\_) {

33  this->stepsize\_adaptation\_.learn\_stepsize(this->nom\_epsilon\_, s.accept\_stat());

34  this->update\_L\_();

35  }

36

37  return s;

38

39  }

40

41  void disengage\_adaptation() {

42  base\_adapter::disengage\_adaptation();

43  this->stepsize\_adaptation\_.complete\_adaptation(this->nom\_epsilon\_);

44  }

45

46  };

47

48  } // mcmc

49

50 } // stan

51

52

53 #endif

stan::mcmc::stepsize\_adaptation::complete\_adaptation

void complete\_adaptation(double &epsilon)

**Definition:** stepsize\_adaptation.hpp:57

unit\_e\_static\_hmc.hpp

stan::mcmc::sample::accept\_stat

double accept\_stat() const

**Definition:** sample.hpp:46

stan::mcmc::base\_adapter::adapt\_flag\_

bool adapt\_flag\_

**Definition:** base\_adapter.hpp:21

stan::mcmc::stepsize\_adaptation::learn\_stepsize

void learn\_stepsize(double &epsilon, double adapt\_stat)

**Definition:** stepsize\_adaptation.hpp:37

stan::mcmc::adapt\_unit\_e\_static\_hmc::~adapt\_unit\_e\_static\_hmc

~adapt\_unit\_e\_static\_hmc()

**Definition:** adapt\_unit\_e\_static\_hmc.hpp:26

stan::mcmc::adapt\_unit\_e\_static\_hmc::transition

sample transition(sample &init\_sample)

**Definition:** adapt\_unit\_e\_static\_hmc.hpp:28

stan::mcmc::adapt\_unit\_e\_static\_hmc::adapt\_unit\_e\_static\_hmc

adapt\_unit\_e\_static\_hmc(M &m, BaseRNG &rng, std::ostream \*o=&std::cout, std::ostream \*e=0)

**Definition:** adapt\_unit\_e\_static\_hmc.hpp:22

stan::mcmc::adapt\_unit\_e\_static\_hmc

**Definition:** adapt\_unit\_e\_static\_hmc.hpp:17

stan::mcmc::adapt\_unit\_e\_static\_hmc::disengage\_adaptation

void disengage\_adaptation()

**Definition:** adapt\_unit\_e\_static\_hmc.hpp:41

stan::mcmc::base\_hmc< M, unit\_e\_point, unit\_e\_metric, expl\_leapfrog, BaseRNG >::nom\_epsilon\_

double nom\_epsilon\_

**Definition:** base\_hmc.hpp:142

stan::mcmc::base\_adapter::disengage\_adaptation

virtual void disengage\_adaptation()

**Definition:** base\_adapter.hpp:15

stepsize\_adapter.hpp

stan::mcmc::unit\_e\_static\_hmc

**Definition:** unit\_e\_static\_hmc.hpp:18

stan::math::e

double e()

Return the base of the natural logarithm.

**Definition:** constants.hpp:86

stan::mcmc::stepsize\_adapter::stepsize\_adaptation\_

stepsize\_adaptation stepsize\_adaptation\_

**Definition:** stepsize\_adapter.hpp:23

stan::mcmc::stepsize\_adapter

**Definition:** stepsize\_adapter.hpp:11

stan::mcmc::base\_static\_hmc< M, unit\_e\_point, unit\_e\_metric, expl\_leapfrog, BaseRNG >::transition

sample transition(sample &init\_sample)

**Definition:** base\_static\_hmc.hpp:28

stan::mcmc::base\_static\_hmc< M, unit\_e\_point, unit\_e\_metric, expl\_leapfrog, BaseRNG >::update\_L\_

void update\_L\_()

**Definition:** base\_static\_hmc.hpp:113

stan::mcmc::sample

**Definition:** sample.hpp:13


---

[ Stan Home Page ]

*© 2011–2014,
Stan Development Team.*
